# Weight change trajectories in Aboriginal and Torres Strait islander Australians after kidney transplantation: a cohort analysis using the Australia and New Zealand Dialysis and Transplant registry (ANZDATA)

**DOI:** 10.1186/s12882-019-1411-1

**Published:** 2019-06-25

**Authors:** Sandawana William Majoni, Shahid Ullah, James Collett, Jaquelyne T. Hughes, Stephen McDonald

**Affiliations:** 1 Department of Nephrology, Division of Medicine, Royal Darwin Hospital, P.O. Box 41326, Casuarina, Darwin, Northern Territory Australia; 2 Flinders University and Northern Territory Clinical School, Royal Darwin Hospital Campus, Darwin, Australia; 3 Menzies School of Health Research Charles Darwin University, Darwin, NT Australia; 4 Australia and New Zealand Dialysis and Transplant (ANZDATA) Registry, SA Health and Medical Research Institute, Adelaide, Australia; 5 Central Northern Adelaide Renal and Transplantation Services, Royal Adelaide Hospital, Adelaide, Australia; 6 Adelaide Medical School, Faculty of Health and Medical Sciences, The University of Adelaide, Adelaide, South Australia Australia

**Keywords:** End stage kidney disease, Transplantation, Body mass index, Weight change trajectory, Aboriginal and Torres Strait islander Australians, Outcomes

## Abstract

**Background:**

Weight change post-kidney transplantation and its associations in Aboriginal and Torres Strait Islander Australians, a group known to have poor patient and graft outcomes, are unknown. Weight change based on body mass index in Aboriginal and Torres Strait Islander Australian recipients was compared to non- indigenous recipients.

**Methods:**

We performed a cohort analysis of data from the Australia and New Zealand Dialysis and Transplant Registry for first deceased donor kidney transplant recipients between 1995 and 2014 in Australia. Weight change post-kidney transplantation was analysed by recipient ethnicity using multivariate mixed effect linear regression analysis.

**Results:**

There were 343 (5.24%) Aboriginal and Torres Strait Islander Australian kidney transplants recipients from a total of 6550 recipients. They had higher pre-transplant BMI (*p* < 0.001), higher rates of current smokers (*p* < 0.001), diabetes (*p* < 0.001), coronary artery disease (*p* < 0.001), cerebrovascular disease (*p* = 0.011) and peripheral vascular disease (*p* = 0.013), ≥4 HLA mismatches (*p* < 0.001), graft loss (*p* < 0.001), mortality (*p* < 0.001) and rejection rates (*p* < 0.001). Weight increased in the first 2 years post-transplantation in both Aboriginal and Torres Strait Islander Australians and non-indigenous Australians. After adjusting for the baseline differences, weight change diverged significantly at 6, 12 and 24 months. The difference was most marked between 6 and 12 months. When stratified by pre-transplantation BMI, all groups except underweight reflected this pattern. Normal weight and obese Aboriginal and Torres Strait Islander Australian recipients had substantial increase at 12 and 24 months and overweight at 6, 12 and 24 months.

The difference in BMI trajectories between Aboriginal and Torres Strait Islander Australians and non- indigenous Australian transplant recipients persisted after adjustment in multivariate mixed effect linear regression analysis.

**Conclusions:**

Post-kidney transplantation weight gain in this high-risk population is substantial and greater than in non-indigenous Australians. Further studies should assess the effect of treatment factors and weight gain on transplant and recipient outcomes.

## Background

Weight gain in non-indigenous kidney transplant recipients is common and predicts outcomes for patient and graft survival [1–3]. Weight changes and their associations in Aboriginal and Torres Strait Islander Australians after kidney transplantation are unknown. In the non-indigenous population, post-transplantation weight gain has been reported at between 10 and 35%, with the majority of the weight gain occurring in the first 12 months post-transplanation [4–7].

Significant weight gain post-kidney transplantation in non-indigenous patients is associated with a number of negative outcomes including post-transplant hypertension [8], chronic allograft nephropathy [2, 9], new onset diabetes after transplanationt [9], and death with a functioning graft [1, 5, 10–12]. There is a paucity of similar data in Aboriginal and Torres Strait Islander transplant recipients; a group with known inferior transplant outcomes [13], lower transplantation rates [14] and high burden of end stage kidney disease requiring renal replacement therapy [15].

In general, Aboriginal and Torres Strait Islander Australian adults are 2.6 times as likely as non-indigenous adults to smoke daily, 1.2 to 1.6 times as likely to be overweight or obese, and 1.2 times as likely to have high blood pressure [16, 17]. They have higher rates of cardiovascular disease, are 3.5 times as likely to have diabetes and 2 to 4 times as likely to have biomedical signs of chronic kidney disease (CKD) [16, 17].

There are many postulated associations with the tendency to increased weight and obesity in Aboriginal and Torres Strait Islander Australians which include various geographical, social, economic and infrastructure factors that affect food choices and availability [17, 18].

Overweight and obesity are casually linked with cardiovascular disease, including coronary heart disease, stroke, heart failure and their associated risk factors [19]. It is presently unknown if Aboriginal and Torres Strait Islander Australians gain weight post-transplantation. If this occurs, the amount, timing, distribution and metabolic effects and impact on patient and graft survival are unclear.

We aimed to examine the weight change trajectories of body mass index (BMI) in Aboriginal and Torres Strait Islander kidney transplant recipients and compare with their non-indigenous counterparts.

## Methods

### Study design

We performed a cohort analysis using the Australia and New Zealand Dialysis and Transplant Registry which collects data on all kidney transplant recipients from hospitals across Australia and New Zealand.

### Participants and data extraction

ANZDATA collects observational data on all patients receiving chronic renal replacement therapy in Australia. All data are collected and submitted to ANZDATA by the treating nephrologist or renal health team at each local site. Data was extracted on all deceased donor recipients in Australia aged ≥18 years, Aboriginal and Torres Strait Islander and non-indigenous, who received a single organ first kidney transplant over the 20 years from 1995 to 2014.

Data extracted included age at transplantation, gender, ethnicity (Aboriginal and Torres Strait Islanders and non-indigenous), transplant era (1995–1999, 2000–2004, 2005–2009, 2010–2014), the number of human leukocyte antigen (HLA) mismatches, dialysis modality and vintage, and recipient comorbidities including hypertension, cardiovascular and peripheral vascular disease, diabetes mellitus, smoking status, BMI (see below), and donor-related factors (donor age and gender) and the duration of total ischemia, delayed graft function, prednisolone dose and rejection. The detailed definitions and measurement of each variable and characteristics can be found in the ANZDATA survey instruction material at http://www.anzdata.org.au/forms/2013SurveyInstructions_v2014.pdf.

### Examining changes in body mass index

BMI was calculated from the quotient of the weight and the square of the height at the time of transplantation. We examined rates of changes in BMI from transplantation using weights at 3,6,12 and 24 months post-transplantation. Patterns were compared between Aboriginal and Torres Strait Islander and non-indigenous recipients. Further analyses stratified recipients based on BMI at time of transplantation into: underweight (BMI < 18.5 kg/m^2^), normal weight (BMI 18.5–24.9), overweight (BMI 25–29.9), and obese (BMI ≥ 30) [20].

### Statistical analysis

Descriptive statistics were expressed as mean and standard deviation for discrete and continuous measures. Percentages were reported for categorical variables. Independent sample T-test and Chi-square analyses were conducted to determine the differences of recipients’ baseline characteristics between Aboriginal and Torres Strait Islander and non-indigenous groups.

A multivariate mixed effect linear regression model was applied to fit linear mixed models of weight trajectories. As the outcome (weight) occurred for each participant at repeated time points, a mixed (random) effect models was used to account for the hierarchical structure of the data. The fixed effects were the group effect (Indigenous vs non-indigenous groups), time effect (Baseline, 3, 6, 12 and 24 months), and group x time interaction and were analogous to regression coefficients. The random effect represented the estimated variability in the intercept (random intercept for patients) to account for repeated measurements. The model was adjusted by the baseline measure of outcome variable. Maximum likelihood estimate procedure was used to compare the significant differences of BMI over time and between Aboriginal and Torres Strait Islander and non-indigenous groups. Univariate models were first used to explore the association between Indigenous status and the change in BMI. A multivariate modelling approach was then undertaken by adding variables considered clinically important or statistically significant from the univariate model, to adjust for confounding. A series of models were undertaken by adding and subtracting variables, with changes in model fit assessed by log likelihood to choose the final multivariate model. The two-sided test was performed for all analysis and the level of significance was set at *p* < 0.05. All analyses were performed using STATA software version 14.1 (StataCorp 2014©1985–2014 StataCorp LP).

The clinical and research activities being reported are consistent with the Principles of the Declaration of Istanbul as outlined in the ‘Declaration of Istanbul on Organ Trafficking and Transplant Tourism’.

## Results

### Baseline characteristics

There were 6550 first time, single organ deceased donor kidney transplant recipients in Australia from 1995 to 2014. 343 (5.24%) recipients were Aboriginal and Torres Strait Islander Australians (Fig. 5). The differences between the two groups are shown in Table 1. Aboriginal and Torres Strait Islander Australians had higher baseline BMI at the time of transplantation, higher rates of current smokers, diabetes mellitus, coronary artery disease, cerebrovascular disease and peripheral vascular disease. Aboriginal and Torres Strait Islander recipients had longer median total ischaemia time, higher rates of ≥4 human leucocyte antigen (HLA) mismatches, longer dialysis vintage, higher rates of graft loss and higher mortality rate. Rates of rejection, delayed graft function and prednisolone dose were higher among the Aboriginal and Torres Strait Islander vs non-indigenous recipients.

**Table 1:** Recipient’s baseline characteristics based on Indigenous status

Characteristic	Non-Indigenous* N = 6207 (94.8%)	Indigenous* N = 343 (5.2%)	*P* value**
Age (years) at transplantation, Mean (SD)	50 (12.3)	45.7 (10.1)	< 0.001
Male, n (%)	3904 (62.9)	185 (53.9)	< 0.001
Current Smoker, n (%)	746 (12)	94 (27.4)	< 0.001
Diabetes mellitus, n (%)	911 (14.7)	170 (49.6)	< 0.001
Coronary artery disease, n (%)	1109 (17.9)	98 (28.6)	< 0.001
Cerebrovascular disease, n (%)	326(5.3)	29 (8.5)	0.011
Peripheral vascular disease, n (%)	596 (9.6)	47 (13.7)	0.013
Total ischaemia time, Median (IQR)	13.0 (10.0, 16.0)	16.0 (12.0, 20.0)	< 0.001
Mismatch n (%)			< 0.001
0	259 (4.2)	1 (0.3)	
1	685 (11.0)	20 (5.8)	
2	1466 (23.6)	34 (9.9)	
3	1078 (17.4)	33 (9.6)	
4	960 (15.5)	58 (16.9)	
5	1126 (18.1)	111 (32.4)	
6	633 (10.2)	86 (25.1)	
Transplant era, n (%)			0.71
1995–1999	1254 (20.2)	64 (18.7)	
2000–2004	1309 (21.1)	73 (21.3)	
2005–2009	1386 (22.3)	85 (24.8)	
2010–2014	2258 (36.4)	121 (35.3)	
Dialysis vintage, n (%)			< 0.001
Pre-emptive	230 (3.7)	5 (1.5)	
< 1 year	573 (9.2)3	18 (5.2)	
1–5 years	805 (61.3)	206 (60.1)	
> 5 years	1599 (25.8)	114 (33.2)	
BMI at time of transplant (kg/m2), Mean (SD)	26.3 (4.7)	27.6 (5.7)	< 0.001
BMI at time of transplant (kg/m2), n (%)			< 0.001
< 18.5	176 (2.8)	9 (2.6)	
18.5–24.9	2415 (38.9)	103 (30.0)	
25–29.9	2192 (35.3)	113 (32.9)	
> 30	1281 (20.6)	104 (30.3)	
Dialysis modality before transplantation			< 0.001
HD	4597 (74.1)	294 (85.7)	
PD	1588 (25.6)	49 (14.3)	
Rejection, n (%)	1531 (24.7)	118 (34.4)	< 0.001
Delayed graft function, n (%)	1554 (25.0)	123 (35.9)	< 0.001
Prednisolone doses (mg), n (%)			< 0.01
0	427 (6.9)	31 (9.0)	
1–4.99	230 (3.7)	11 (3.2)	
5–6.99	2039 (32.9)	81 (23.6)	
7–9.99	1062 (17.1)	45 (13.1)	
10+	1520 (24.5)	103 (30.0)	
Donor age (years)	44.2 (17.5)	44 (17.5)	0.77
Donor male, n (%)	3575 (57.6)	201 (58.6)	0.72

*Some characteristics do not add up to 100% due to missing cases for that characteristic

**P values are based on Chi-square test for percentages, independent sample t-test for means and Mann Whitney U test for medians

*HD* Haemodialysis, *PD* Peritoneal Dialysis

There were no significant differences in the overall immunosuppression.

### Weight change trajectories: comparison between aboriginal and Torres Strait islander and non-indigenous recipients

#### Weight change after adjusting for baseline differences

**Fig. 1:**
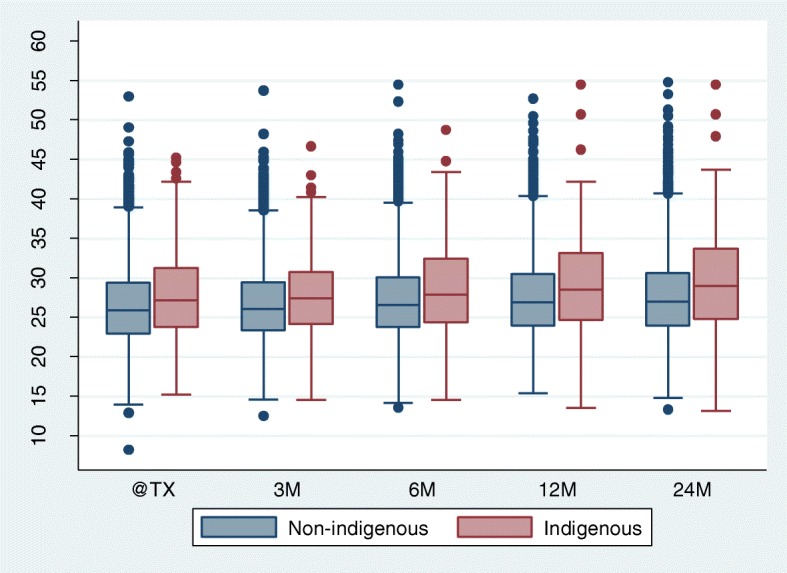
Box plot representing observed BMI at transplantation and 3, 6, 12 and 24 months post-transplantation in Aboriginal and Torres Strait Islander vs Non-Indigenous Australians. Data shown are median, inter-quartile range, minimum and maximum BMI

**Fig. 2:**
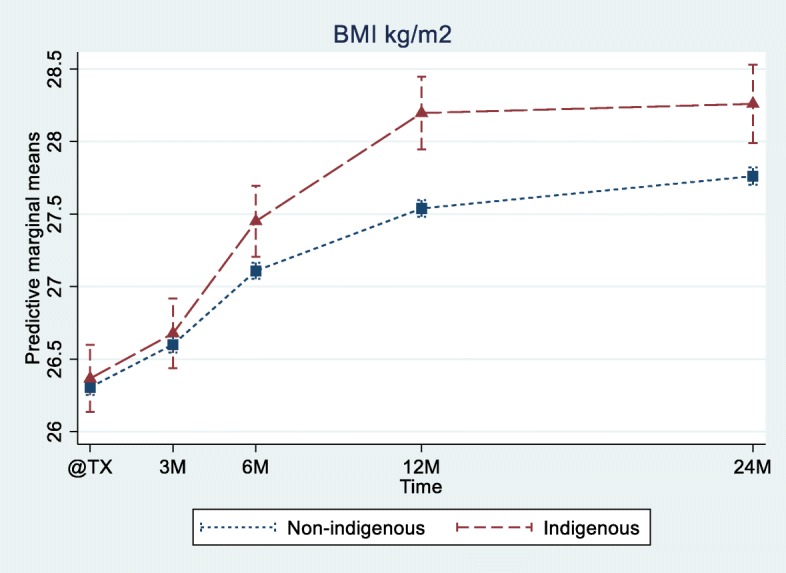
Weight trajectories in Aboriginal and Torres Strait Islander vs Non-Indigenous transplant recipients over the first 2 years after transplant. The lines show the estimated marginal means from mixed effect linear model at 0, 3, 6, 12 and 24 months

Median BMI increased significantly in the first 2 years after transplantation in both groups (Fig. 1). Using a mixed effect model to adjust for the baseline differences, both groups had similar weight change trajectories for the first 3 months but then diverged significantly, with a positive change of 0.34 kg/m^2^ (0.09 kg/m^2^–0.59 kg/m^2^), 0.66 kg/m^2^ (0.40 kg/m^2^–0.92 kg/m^2^) and 0.50 kg/m^2^ (0.22 kg/m^2^–0.78 kg/m^2^) at 6, 12 and 24 months respectively in the Aboriginal and Torres Strait Islander group mean BMI compared with the non-indigenous group (Fig. 2). The difference in the rate of BMI change between the two groups was most marked between 6 and 12 months post-transplantation.

#### Weight change by baseline BMI strata at the time of transplantation

**Fig. 3:**
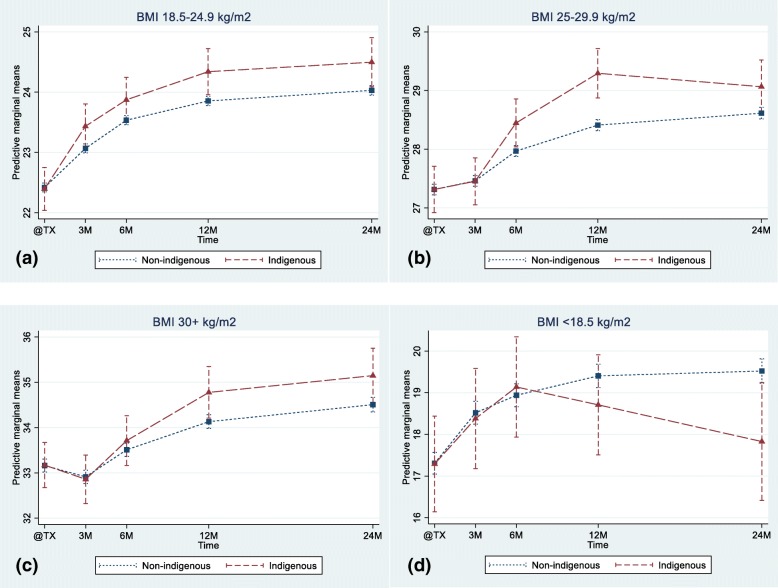
Weight trajectories over the first 2 years post Kidney transplantation in Aboriginal and Torres Strait Islander vs Non-Indigenous transplant recipients and based on BMI category at time of transplant

When stratified by BMI at the time of transplantation, all groups except underweight patients reflected this overall pattern of increasing BMI. Compared with the change in their non-indigenous counterparts, normal weight Aboriginal and Torres Strait Islanders recipients had significantly increased mean BMI gain at 12 and 24 months (Fig. 3a), overweight Aboriginal and Torres Strait Islander recipients had significantly elevated BMI at 6, 12 and 24 months (Fig. 3b), while obese Aboriginal and Torres Strait Islander recipients had increased mean BMI that reached statistical significance at 12 and 24 months (Fig. 3c). However, underweight Aboriginal and Torres Strait Islander recipients had similar BMI changes up to 6 months post-transplant, but at 24 months, had a significant lower BMI compared with the non-indigenous group (Fig. 3d).

#### Weight change after adjusting for confounders and pre-existing risk factors at the time of transplantation

As shown in Table 2, using multivariate mixed effect models accounting for confounders shown in Table 1, the difference in weight trajectories between Aboriginal and Torres Strait Islander and non-indigenous transplant recipients persisted with a significant difference favouring a higher BMI in the Aboriginal and Torres Strait Islander group at 6, 12 and 24 months. A significant association with BMI was also observed for younger recipient and donor, recipients with coronary artery disease and longer total ischemia time.

**Table 2:** Marginal mean change in BMI (Multivariate mixed effect model) between Indigenous and Non-Indigenous groups with confounders

Variables	Univariate Model [1]			Multivariate Model 1^2^			Multivariate Model 2^3^		
	Coefficient [4] (95% CI)	RE % [5]	*P* value	Coefficient^4^ (95% CI)	RE % [5]	*P* value	Coefficient^4^ (95% CI)	RE % [5]	*P* value
Indigenous status									
Non-indigenous	Reference	–	–	Reference	–	–	Reference	–	–
Indigenous	0.06 (− 0.18–0.30)	6.2	0.618	− 0.05 (− 0.29–0.19)	−4.9	0.687	−0.04 (− 0.32–0.23)	−4.1	0.765
Time post-transplant (months)									
0	Reference	–	–	Reference	–	–	Reference	–	–
3	0.29 (0.24–0.35)	34.2	< 0.001	0.30 (0.24–0.35)	34.7	< 0.001	0.33 (0.27–0.39)	39.2	< 0.001
6	0.80 (0.75–0.86)	123.2	< 0.001	0.81 (0.75–0.86)	123.9	< 0.001	0.84 (0.78–0.90)	132.6	< 0.001
12	1.23 (1.18–1.29)	243.1	< 0.001	1.24 (1.18–1.29)	243.1	< 0.001	1.26 (1.20–1.32)	251.4	< 0.001
24	1.46 (1.40–1.51)	328.7	< 0.001	1.46 (1.40–1.52)	328.7	< 0.001	1.48 (1.41–1.54)	337.6	< 0.001
BMI at time of transplantation	0.95 (0.94–0.96)	159.5	< 0.001	0.96 (0.96–0.97)	159.5	< 0.001	0.97 (0.96–0.98)	162.8	< 0.001
Indigenous status x time (months)									
post-transplant (interaction) [6]									–
Indigenous and 3	0.02 (−0.23–0.26)	1.7	0.894	0.01 (−0.24–0.26)	0.8	0.950	− 0.003 (− 0.27–0.26)	−0.3	0.98
Indigenous and 6	0.28 (0.03–0.53)	32.5	0.029	0.27 (0.02–0.53)	31.4	0.034	0.34 (0.07–0.61)	40.9	0.012
Indigenous and 12	0.60 (0.34–0.86)	81.8	< 0.001	0.59 (0.33–0.85)	80.5	< 0.001	0.61 (0.34–0.88)	84.1	< 0.001
Indigenous and 24	0.44 (0.16–0.72)	55.0	< 0.01	0.43 (0.15–0.71)	54.0	< 0.01	0.45 (0.16–0.74)	57.1	< 0.01
Recipient age, years				−0.03 (− 0.033--.026)	−2.9	< 0.001	− 0.03 (− 0.04--0.03)	−3.1	< 0.001
Gender									
Male				Reference	–	–	Reference	–	–
Female				0.20 (0.11–0.28)	21.9	< 0.001	0.21 (0.12–0.31)	23.6	< 0.001
Current smoking status									
No				Reference	–	–	Reference	–	–
Yes				0.09 (−0.03–0.21)	9.5	0.151	0.08 (− 0.05–0.21)	8.3	0.245
Diabetes Mellitus									
No				Reference	–	–	Reference	–	–
Yes				0.02 (−0.10–0.14)	2.0	0.748	0.01 (−0.12–0.15)	1.4	0.845
Coronary artery disease									
No				Reference	–	–	Reference	–	–
Yes				0.17 (0.05–0.29)	18.6	< 0.01	0.18 (0.05–0.32)	20.1	< 0.01
Cerebrovascular disease									
No				Reference	–	–	Reference	–	–
Yes				−0.16 (−0.35–0.03)	−14.7	0.095	−0.18 (− 0.39–0.03)	−16.2	0.096
Peripheral vascular disease									
No				Reference	–	–	Reference	–	–
Yes				−0.04 (−0.20–0.11)	−4.2	0.585	−0.05 (− 0.22–0.13)	−4.5	0.600
Total ischemia time				−0.02 (− 0.03--.01)	− 1.8	< 0.001	− 0.02 (− 0.03–0.02)	−1.7	< 0.001
HLA antigen mismatch				− 0.02 (− 0.04–0.01)	−1.6	0.222	−0.01 (− 0.04–0.02)	−1.1	0.422
Dialysis vintage									
Pre-emptive				Reference	–	–	Reference	–	–
< 1 year				−0.01 (−0.82–0.79)	−1.2	0.977	0.01 (−0.87–0.89)	1.2	0.978
1–5 years				−0.04 (− 0.84–0.75)	−4.4	0.912	−0.01 (− 0.89–0.86)	−1.1	0.980
> 5 years				−0.14 (− 0.94–0.66)	−12.8	0.737	−0.09 (− 0.97–0.79)	−8.3	0.846
Transplantation era									
1995–1999				Reference	–	–	Reference	–	–
2000–2004				−0.05 (−0.18–0.07)	−5.2	0.415	−0.08 (− 0.22–0.05)	− 8.0	0.235
2005–2009				0.07 (−0.06–0.20)	7.4	0.283	0.04 (−0.10–0.18)	4.2	0.570
2010–2014				0.14 (0.01–0.27)	15.2	0.029	0.12 (−0.03–0.26)	12.3	0.121
Donor age				−0.01 (− 0.01--0.004)	−0.7	< 0.001	− 0.01 (− 0.01--0.004)	−0.7	< 0.001
Donor gender									
Male				Reference	–	–	Reference	–	–
Female				0.02 (−0.06–0.10)	1.9	0.663	0.02 (−0.07–0.11)	2.1	0.651
Rejection									
No							Reference	–	–
Yes							−0.10 (−0.20–0.004)	−9.6	0.058
Delayed graft function									
No							Reference	–	–
Yes							−0.17 (− 0.28--0.05)	−15.3	< 0.001
Prednisolone doses quarterly (mg)									
0							−0.26 (− 0.44--0.09)	−22.9	< 0.001
1–4.99 mg							0.20 (−0.03–0.43)	29.7	0.08
5–6.99 mg							Reference	–	–
7–9.99 mg							−0.03 (−0.16–0.09)	−3.0	0.59
10+ mg							−0.11 (− 0.22–0.01)	−10.4	0.06
Constant/intercept	1.22 (0.98–1.46)	< 0.001	–	2.87 (2.02–3.73)	–	< 0.001	2.74 (1.80–3.69)	–	< 0.001

1. Univariate model includes indigenous status, time post transplants and interaction between indigenous status and time post transplants

2. Multivariate model 1 includes variables from model 1 plus recipient’s age, gender, smoking status, diabetes mellitus, comorbidities, ischemia time, HLA mismatches, dialysis vintage, transplant ERA, Donor’s age and gender

3. Multivariate model 2 includes variables from multivariate model 1 plus rejection, delayed graft function, and prednisolone doses

4. Marginal mean change in BMI (kg/m^2^)

5. RE% corresponding to a coefficient expressed as expected percentage change in BMI, calculated as (exp(coefficient)-1)*100

6. In addition to main effects (indigenous status and time), the interaction term (indigenous status x time) explains the BMI change between Indigenous and non-indigenous groups by different times post transplants

#### Weight change after adjusting for post-transplantation factors

When the post-transplantation factors (rejection, delayed graft function and prednisolone dose) were added (model 2, Table 2), the same factors remained significant in addition to the significant association with delayed graft function and prednisolone dose. Surprisingly, for differences between Aboriginal and Torres Strait Islander and non-indigenous Australians the occurrence of rejection had no statistically significant association with BMI. However, after stratifying by rejection and analysing the BMI trajectories between Aboriginal and Torres Strait Islander and non-indigenous group, the BMI was significantly higher for the occurrence of rejection in the Aboriginal and Torres Strait Islander patients (compared to non-indigenous) than for the patients who did not have any rejection (Table 4). These post-transplantation factors (delayed graft function and prednisolone dose) may partly explain the observed weight trajectories post-transplantation.

**Fig. 4:**
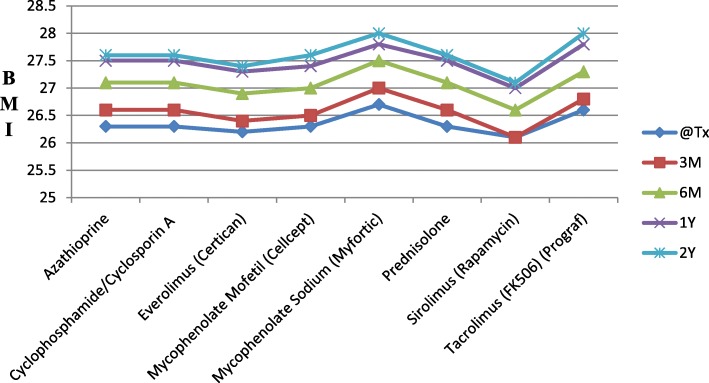
BMI changes by immunosuppression regimens over the study period

There was no overall trend in the BMI change post- transplantation across immunosuppression regimens, although there was a non-statistically significant tendency to higher BMI in those on tacrolimus and mycophenolate sodium (Table 3 and Fig. 4).

**Table 3:** Immunosuppression regimens received during the course of the study and related BMI changes

Immunosuppression regimens	Number (Percent)	Mean BMI (kg/m^2^)				
		@Tx	3 M	6 M	12 M	24 M
Azathioprine	734 (11%)	26.3	26.6	27.1	27.5	27.6
Cyclosporin A	3027 (46%)	26.3	26.6	27.1	27.5	27.6
Everolimus	84 (1%)	26.2	26.4	26.9	27.3	27.4
Mycophenolate Mofetil	4563 (70%)	26.3	26.5	27.0	27.4	27.6
Mycophenolate Sodium	1041 (16%)	26.7	27.0	27.5	27.8	28.0
Prednisolone	6527 (100%)	26.3	26.6	27.1	27.5	27.6
Sirolimus	246 (4%)	26.1	26.1	26.6	27.0	27.1
Tacrolimus	3530 (54%)	26.6	26.8	27.3	27.8	28.0

Key: @tx; at the time of transplantation, 3 M; at 3 months post-transplantation, 6 M; at 6 months post-transplantation, 12 M; at 12 month post-transplantation, 24 M; at 24 months post-transplantation

There was no statistically significant cohort effect (that is, being transplanted in the periods 1995–1999, 2000–2004, 2005–2009, 2010–2014) on weight gain in the first 24 months post-kidney transplantation between the two groups.

## Discussion

This is the first study to assess weight change trajectories among Aboriginal and Torres Strait Islander Australian kidney transplant recipients, a group with high background rates of metabolic disease, poorer access to transplantation and post-transplantation outcomes. The main findings are: 1) there are substantial differences between Aboriginal and Torres Strait Islander and non-indigenous recipients in the baseline clinical characteristics potentially associated with weight gain and cardiovascular risk post-kidney transplantation 2) Aboriginal and Torres Strait Islander Australian kidney transplant recipients had both higher prevalence of overweight and higher absolute BMI preceding transplantation than non-indigenous patients, 3) although there is an increase in weight gain in both groups in the first 2 years, this increase is substantially higher in Aboriginal and Torres Strait Islander Australians, 4) Predictors of weight gain included young age, female gender, longer total ischemia time, cardiovascular disease, older donor age, delayed graft function and prednisolone dose and 5) although there is an association between post transplantation factors (delayed graft function and prednisolone dose) and BMI, the association with rejection was only apparent after stratifying BMI trajectories between Aboriginal and Torres Strait Islander and non-indigenous group by rejection (rejection or no rejection) (Table 4).

**Table 4:** Marginal mean change in BMI (mixed effect model) rejection and non-rejection group

	No rejection (*n* = 4901)			Rejection (*n* = 1649)		
	Coefficient (95% CI)	RE %	*P* value	Coefficient (95% CI)	RE %	*P* value
Indigenous status						
Non-indigenous	Reference	–	–	Reference	–	–
Indigenous	0.07 (−0.22–0.36)	7.3	0.651	0.04 (− 0.37–0.47)	4.1	0.839
Time post-transplant (months)	Reference	–	–	Reference	–	–
0	0.32 (0.26–0.38)	37.7	< 0.001	0.22 (0.10–0.33)	24.6	< 0.001
3	0.84 (0.78–0.91)	131.6	< 0.001	0.69 (0.57–0.81)	99.4	< 0.001
6	1.26 (1.20–1.33)	252.5	< 0.001	1.15 (1.03–1.27)	215.8	< 0.001
12	1.47 (1.40–1.53)	334.9	< 0.001	1.43 (1.30–1.55)	317.9	< 0.001
24						
Indigenous status x time (months) post-transplant (interaction)						
Indigenous and 3	0.07 (−0.23–0.36)	7.3	0.670	−0.04 (− 0.48–0.41)	−3.9	0.868
Indigenous and 6	0.20 (−0.11–0.51)	22.1	0.200	0.47 (0.02–0.92)	60.0	0.042
Indigenous and 12	0.39 (0.08–0.71)	47.7	0.015	0.97 (0.51–1.43)	163.8	< 0.001
Indigenous and 24	0.29 (−0.05–0.63)	33.6	0.097	0.69 (0.20–1.18)	99.4	< 0.01

RE% corresponding to a coefficient expressed as expected percentage change in BMI, calculated as (exp(coefficient)-1)*100

The increase in weight observed in the first 24 months in both groups is consistent with post-transplantation weight gain seen in non-indigenous patients in other populations [5]. The striking difference in the results of this study is the higher weight gain in Aboriginal and Torres Strait Islander Australians. This may be explained by the higher baseline BMI, which is consistent with findings in other studies comparing BMI between the two groups, [19] and other substantial differences between Aboriginal and Torres Strait Islander and non-indigenous Australians in other baseline risks of obesity at transplantation (Table 1) [15, 21].

Studies of post-transplantation outcomes in Aboriginal and Torres Strait Islander Australians suggest the poorer outcomes compared to non-indigenous Australians are predominantly due to infectious causes with rates of other causes such as cardiovascular disease and cancers appearing to be similar [13, 22]. These studies are limited by design issues (mostly retrospective analyses) and small sample sizes.

In some populations, high BMI and weight gain post-kidney transplantation is associated with post-transplant hypertension, diabetes mellitus, ischemic heart disease and poor graft and patient outcomes [9]. However, other studies have shown no association with patient or graft outcomes [23] . Some of these studies suggested that only demographic factors rather than treatment factors may contribute to the risk of weight gain post-kidney transplantation [5]. Young age, black ethnicity and female gender were associated with more weight gain in these studes [5]. Results from other studies conflicted these findings [24] . Our results agree with other studies suggesting an association of these factors with weight gain [5].

The higher BMI in Aboriginal and Torres Strait Islander Australians at transplantation and the higher weight gain throughout the first 2 years post- transplantation in all BMI categories (except for low BMI) can be explained by several possible factors as outlined above. The higher baseline BMI may reflect of higher rates of obesity in Aboriginal and Torres Strait Islander Australians [25] (consistent with previous data [15]) which partly explains the increased risk of CKD observed in this population. There is evidence to support an increased prevalence of the metabolic syndrome among Aboriginal and Torres Strait Islander Australians which positively correlates with prevalence rates of CKD [26]. From the results of a recent study, another potential explanation for the high BMI at the time of kidney transplantation and hence the post-transplantation weight gain is the difference in body composition and fat-free mass in Aboriginal and Torres Strait Islander Australians versus non-ndigenous Australians [27].

Some studies have shown no relationship between steroid dose and weight gain [8]. Our analysis included the potential effect of steroid use and other treatment factors (Table 2, model 2). Consistent with studies examining outcomes in Aboriginal and Torres Strait Islander Australians, the association of higher rejection rates with Aboriginal and Torres Strait Islander ethnicity may be associated with intermittent bursts of higher steroid dose exposure as treatment for rejection. This requires further exploration as a potential contributor to weight gain [13, 15, 22]. The lack of association with rejection (before stratifying BMI trajectories between Indigenous and non-Indigenous group by rejection) was surprising and needs further exploration. However, this is consistent with another previous study which reported no association [5]. This has further contributed to the uncertainty of this association [1].

The reason for the decrease in weight in the underweight BMI group is unclear. Although there were small numbers in the group (9 recipients), this may reflect either graft failure and return to dialysis or poorer health in that subgroup.

Clues as to why the weight trajectories differ between the two groups despite adjustment for all known confounders may also lie in variables that are not captured in data collected in the registry. Differences in diet, drug pharmacokinetics in different ethnic groups, healthy literacy level and other social factors are all important considerations.

Some studies of Aboriginal and Torres Strait Islander Australians have concluded that BMI may underestimate the cardiometabolic risks associated with overweight and obesity in this population [26–28]. These studies have indicated that due to differences in body fat distribution in Aboriginal and Torres Strait Islander Australians than Non-Indigenous populations, using anthropometric measurements (particularly waist circumference and waist/hip ratio measurements which may improve the cardio metabolic risk assessments than BMI) is a more accurate measure of overweight and obesity [26–28]. Including these anthropometric measurements for potential Aboriginal and Torres Strait Islander transplant recipients may give a further clarity on their value in predicting the weight changes after transplantation.

The weight change trajectories raise questions as to why this difference exists between Aboriginal and Torres Strait Islander and Non-Indigenous kidney transplant recipients. One study from North America reported African-American ethnicity as one of the factors associated with weight gain [29]. Hence, ethnicity may partly explain this difference between Aboriginal and Torres Strait Islander and Non-Indigenous Australians.

A recent clinical trial on reducing weight gain post transplantation by intensive nutritional intervention versus standard nutritional intake reported no benefit in the first year post transplantation [30]. There are other ongoing studies assessing other interventions such as lifestyle changes and lifestyle and dietary changes [31]. Given this lack of evidence, from our results, standard advice on reducing weight would apply to both Aboriginal and Torres Strait Islander and non-indigenous Australians although there is need for stronger emphasis in Aboriginal and Torres Strait Islander Australians.

Our results also raise further questions about weight change trajectory’s impact on graft and patient survival in Aboriginal and Torres Strait Islander Australians, which is beyond the scope of this paper but is highlighted as an area for future research.

**Fig. 5:**
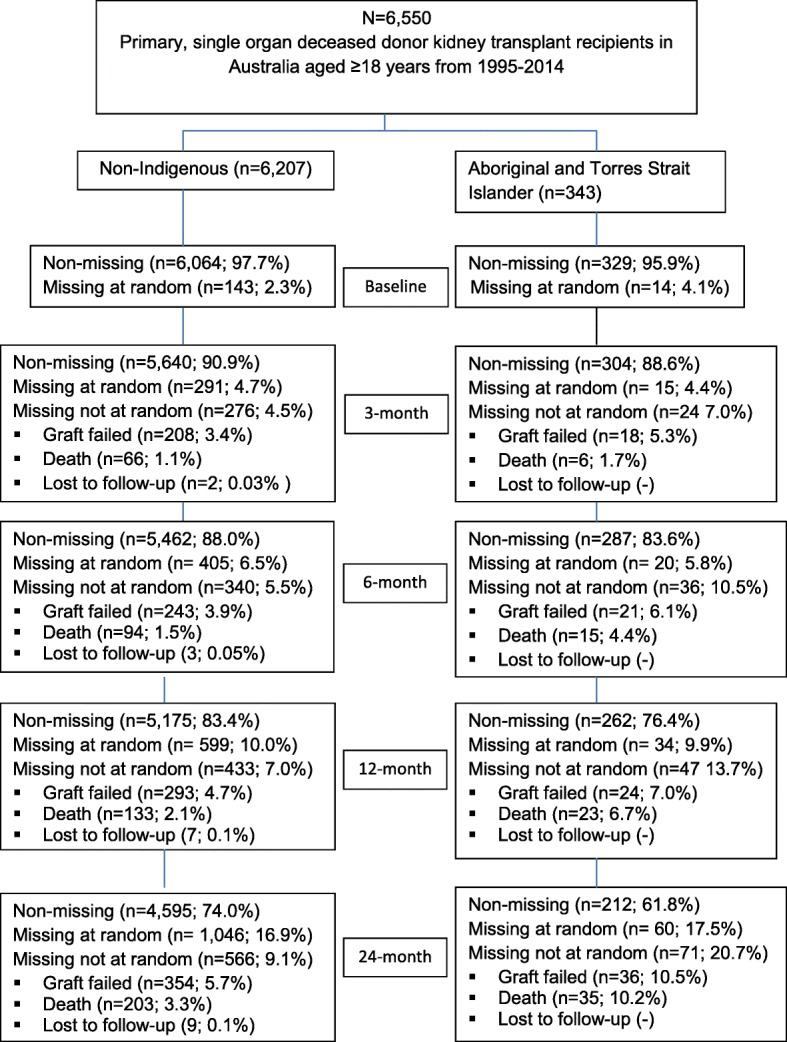
Patient’s flow and missing ness of BMI for baseline, 3, 6, 12, and 24 months

We acknowledge limitations of our study are those inherent with the use of observational data. This includes the limitations inherent in the data collected for registries, for example ANZDATA does not collect information on lifestyle factors. The impact on post-transplant weight gain of pre-existing risk factors will be better assessed in a case-control study. Another limitation was the amount of missing data. The missing data is not entirely at random as at 24 months it was 9.7% due to graft loss or death or lost to follow-up and the missing at random was 16.9% (Fig. 5). However, the patient characteristics and confounders were analyzed by stratification of non-missing and missing data and this was found to be insignificant for almost all the characteristics in the Aboriginal and Torres Strait Islander group. Additionally, it could be argued that questions on the accuracy of BMI as a measure of overweight and obesity in Aboriginal and Torres Strait Islander Australians may affect the accuracy of this comparison as anthropometric measurements are more reliable than BMI in classifying Aboriginal and Torres Strait Islander patients into a particular weight category [28]. However, as this study compares weight change rather than absolute differences, this may not have a significant effect on the difference in weight change observed between the groups.

## Conclusions

Aboriginal and Torres Strait Islander Australians have a higher weight-gain in the first 24 months following kidney transplantation than non-indigenous Australians in all categories of BMI except underweight. Post-transplantation weight-gain did not appear to be explained by transplant rejection. Post-transplantation health and cardio-metabolic risk is vital in promoting this form of renal replacement therapy, especially in populations known to have cardiovascular disease and poor kidney transplant outcomes. We recommend further studies examining; 1) the potential effects of treatment factors such as steroid use and the rates of rejection 2) the potential relationship of this weight gain with transplant and patient outcomes.

### Implications for clinical practice

Equitable access to kidney transplantation is a public health issue [32]. Aboriginal and Torres Strait Islander Australians, who suffer some of the highest rates of CKD in the world, would benefit from kidney transplantation [33]. However, access to kidney transplantation is currently low [14, 34–36]. One of the factors associated with the poor access is poor graft and patient outcomes which are common among Aboriginal and Torres Strait IslanderAustralians [13, 22]. Improving the understanding and management of risk factors for poor outcomes will improve access. Our study demonstrates significant weight gain post transplantation. Determining the effect of this weight gain on graft and patient survival may lead to improvement in outcomes and hence access to kidney transplantation.

## Data Availability

The datasets generated and/or analyzed during the current study are available from the Australia and New Zealand Dialysis and Transplant Registry (ANZDATA) http://www.anzdata.org.au/v1/. Data is available according to ANZDATA data release guidelines.

## Abbreviations

ANZDATA: Australia and New Zealand Dialysis and Transplant Registry
BMI: Body mass index
CKD: Chronic kidney disease
HD: Haemodialysis
HLA: Human leukocyte antigens
PD: Peritoneal dialysis
